# Improving the accuracy of revised cardiac risk index with HbA1C: Hemoglobin ratio (HH ratio) – A retrospective cohort study

**DOI:** 10.3389/fmed.2023.998477

**Published:** 2023-03-24

**Authors:** Yuhe Ke, Nicholas Brian Shannon, Hairil Rizal Abdullah

**Affiliations:** ^1^Department of Anesthesia, Singapore General Hospital, Singapore, Singapore; ^2^Department of General Surgery, Singapore General Hospital, Singapore, Singapore

**Keywords:** revised cardiac risk index (RCRI), risk model, HH ratio, diabetes mellitus, HbA1c (glycated hemoglobin test)

## Abstract

**Background:**

The current Lee’s Revised cardiac risk index (RCRI) was created in 1999. Validation studies have found RCRI to be only moderately discriminant. The “Diabetes Mellitus on insulin” component of the score does not accurately reflect the severity of the disease. A previously studied HbA1C:Hemoglobin ratio shows an improved association with outcomes than individual components alone.

**Study design:**

A retrospective cohort study was performed in diabetic patients undergoing non-cardiac surgery. Ethics approval was obtained. The study compares the predictive value of RCRI and substitution of the “DM on insulin” component with HH ratio for 30- and 90-day mortality, and postoperative acute myocardial injury (AMI) and acute kidney injury (AKI).

**Results:**

A total of 20,099 adult patients were included in the final analysis. The incidence of 30- and 90-day mortality was at 4.2 and 6.5%, respectively. Substitution of HH ratio in RCRI resulted in 687 more patients being in the moderate to high-risk category. The substituted HH-RCRI score had better prediction for 30-day (AUC 0.66 vs. 0.69, *p* < 0.001) and 90-day mortality (AUC 0.67 vs. 0.70, *p* < 0.001), and postoperative AMI (AUC 0.69 vs. 0.71, *p* < 0.001) and AKI (AUC 0.57 vs. 0.62, *p* < 0.001).

**Conclusion:**

Although currently not an universal practice, substitution of “DM on insulin” with HbA1C:Hemoglobin ratio in RCRI score improves the accuracy of the RCRI risk prediction model in diabetic patients going for non-cardiac surgery.

## Highlights

- A ratio of HbA1C over Hemoglobin (HH ratio) looks at the double threat of supply-demand mismatch. It is an objective disease severity marker.
- Substitution of “DM on insulin” in the RCRI prediction model with HH ratio removes subjectivity.
- The substituted RCRI-HH score improves the accuracy of prediction for mortality and postoperative AMI and AKI. This is currently not the standard, universal practice.

## Introduction

Preoperative risk stratification is a strategy to predict postoperative outcomes and potentially alter or optimize comorbidities and modifiable risk factors. One commonly used risk stratification in non-cardiac surgery includes Lee’s Revised Cardiac Risk Index (RCRI) (1). First published in 1999, the score predicts perioperative major adverse cardiovascular events during the surgical hospital admission or within 30 days of surgery. The RCRI identifies the following as predictors of adverse outcomes: past medical history (Ischemic heart disease, congestive heart failure, cerebrovascular disease, and diabetes mellitus on insulin), one laboratory finding (creatinine > 180 umol.l^–1^), and one surgical factor (high-risk surgery). The original study found the scoring to have an area under curve (AUC) analysis of 0.76 in the derived cohort and 0.81 in the validation cohort. Subsequent systematic reviews of 24 studies predicting cardiac risks using RCRI were only found to be moderately discriminating with a median AUC of 0.62 (2).

The component involving diabetes mellitus (DM) on insulin is an indirect marker of poor perioperative glycemic control. Previous work has been hypothesized to be associated with increased oxidative stress and free radical build-up (3). This marker is increasingly replaced by preoperative HbA1C value. It is relatively easy to do, does not require fasting, and represents a more accurate glucose control in the last 3 months. However, the lack of high-grade evidence results in differing guidelines of HbA1C cut-off for preoperative optimization ranging from 7.0 to 8.5% in Asian, European, and US guidelines (4–6). Studies looking at the relationship between HbA1C and postoperative complications have been mixed (7–10).

This could be attributed to the multiple factors which confound the reading of HbA1C. In particular, iron-deficiency anemia results in an over-read HbA1C (11, 12), possibly due to the prolonged average age of circulating erythrocytes in iron deficiency anemia which increases the amount of irreversible glycation of hemoglobin (resulting in higher HbA1C reading) (13). Many studies looking at the association of HbA1C with postoperative outcomes have failed to include hemoglobin in the adjusted analysis for endpoints (7). This may have accounted for the mixed outcomes in the literature.

Preoperative anemia is also a surrogate marker for poor physical status and is associated with increased packed cell transfusions (14), morbidity, and mortality (15). A patient with increased HbA1C and reduced hemoglobin hence has a double threat for poor postoperative outcomes. The increased oxidative stress from poor glycemic control with reduced oxygen delivery from anemia results in a supply-demand mismatch. Given the inverse relationship between the HbA1C and hemoglobin, a ratio of HbA1C over hemoglobin (HH ratio) has been investigated in the cardiac surgery population and found to have a positive relationship with mortality and morbidity (16).

This study looks at the predictive value of substitution of HH ratio with the “Diabetes mellitus on insulin” component of RCRI in diabetic patients going for non-cardiac operations. Outcome measurements include postoperative acute myocardial injury (AMI), acute kidney injury (AKI), and 30- and 90-day mortality outcomes.

## Methodology

Ethics approval was obtained from the SingHealth Centralized Institutional Review Board (CIRB Reference number 2020/2915) prior to the start of the study, which waived the requirement for written informed consent due to the use of de-identified routinely available data. This was a retrospective study done by reviewing the electronic medical records of all the patients who had undergone elective non-cardiac surgery between March 2013 and December 2020 (ClinicalTrials.gov, Identifier NCT05066386).

Patients were included if they were above 18 years old, with a past medical history of diabetes, and had undergone non-cardiac surgery. Both emergency and elective surgeries were included. Patients who did not have a preoperative HbA1C reading within the last 3 month were excluded. If multiple preoperative HbA1C and hemoglobin levels were present, the latest preoperative reading was taken.

The clinical records were sourced from our institution’s clinical information system (Sunrise Clinical Manager (SCM), Allscripts, IL, USA) and stored in the enterprise data repository and analytics system (SingHealth-IHiS Electronic Health Intelligence System). Information from SCM including patient demographics, the urgency of operation, preoperative comorbidities such as ischemic heart disease, congestive heart disease, cerebrovascular diseases, and diabetes were recorded. The last active preoperative medications were extracted. Preoperative blood tests including hemoglobin, HbA1C, and creatinine were recorded. Preoperative HbA1C may either be done as part of the routine follow-up for diabetes mellitus (DM) or ordered by a surgeon or anesthetist as part of preoperative workup when deemed necessary.

Operative details including type and duration of surgery and surgical risk were obtained. The type of surgery was classified as low, intermediate, or high risk based on the European Society of Cardiology and European Society of Anesthesiology non-cardiac surgery risk score (17). Lee’s revised cardiac risk index (RCRI) was collected from the preoperative anesthesia form, which is done as part of the routine preoperative assessment for all patients undergoing surgery.

Postoperative creatinine and high-sensitivity troponin-T were extracted up to 7 days postoperatively. The decision to do these tests is based on clinical indications and surgical team decisions. Creatinine is done as part of the urea, creatinine, and electrolyte panel. In our institution, postoperative Troponin-T is routinely done when there is a suspicion of cardiac events (e.g. Hypotension, arrhythmias), chest pain, or electrocardiogram changes.

### Outcomes measurements

The HH ratio was divided into two groups with a cut-off of 0.7 based on a previous study (16) and analysis of the current cohort based on Youden’s index (18) for best cut-off. Glycemic control was divided into HbA1C < 7.0% and HbA1C ≥ 7.0% (19). Anemia is defined by Hb of <13.0 g/dL for both genders.

Postoperative acute myocardial injury (AMI) is defined by patients who had high-sensitivity troponin-T done with a value of >65 ng.L^–1^ (20) at any point of time up to 7 days postoperatively. Postoperative acute kidney injury (AKI) is defined as KDIGO stage 2 with criteria of >2 times elevation of creatinine from baseline within 7 days in the postoperative period.

The mortality date data in our clinical information system is synced with the data from the National Registry of Diseases Office, ensuring a near-complete all-cause mortality capture. Survival days were calculated from the date of hospital admission to the date of death. The cause of death was not collected. The study is reported in line with the STROCSS criteria 2019 (21).

### Statistical analysis

The missing values for all the other variables generally account for <2% and were replaced with the mode in categorical variables and the median in continuous variables. Variables which had >2% missing values were non-essential to the purpose of the study and were discarded from the analysis.

A univariate linear regression model was performed to determine the correlation of RCRI and substituted RCRI score with outcomes. For categorical outcomes, Poisson regression models were used. The effect size was reported as an odds ratio (OR) and its 95% confidence interval (CI). Bonferroni correction was used to adjust for *p*-value in multivariable regression models.

In the “Substituted HH-RCRI score” group, patients with an HH ratio >0.7 would score a positive point. This replaces the score contributed by DM on insulin.

Sensitivity, specificity, and receiver operator characteristics (ROC) were estimated using ROC analysis. Analysis, statistical computing, and visualization were carried out with R environment version 4.0.5. Delong’s test was used to calculate the significance between the two AUC curves.

Exploratory analysis of the additive value of HH ratio to the existing RCRI score was done and showed that the AUC results were similar or marginally better. Hence, it is not discussed in this study as substitution of HH ratio instead of the addition provided better accuracy.

## Results

**FIGURE 1:**
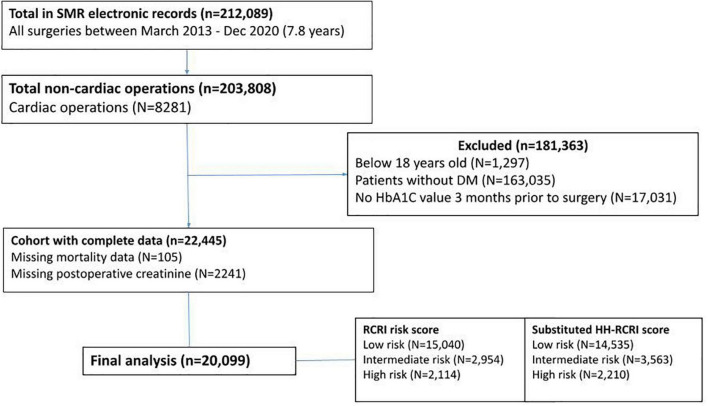
CONSORT diagram. A final number of 20,099 patients were included in the analysis.

A total of 20,099 adult diabetic patients who had undergone non-cardiac surgery were included in the final analysis (Figure 1). The scoring for the different components in RCRI and RCRI with substitution with HH ratio is represented in Table 1.

**TABLE 1:** Components of RCRI score and risk categories, and proposed addition to HH ratio (substituted HH-RCRI score), and proposed risk categories.

RCRI components	RCRI risk categories
1. High risk operations	Low risk: 0−1 Intermediate risk: 2 High risk: 3 and above
2. Ischemic heart disease (IHD)	
3. Heart Failure (HF)	
4. Cerebrovascular disease (CVD)	
5. Elevated serum creatinine > 180 umol.l^–1^	
6. Diabetes mellitus requiring insulin	
**Substitute RCRI’s diabetes mellitus component with HH ratio (Substituted HH – RCRI score)**	**Proposed risk categories**
1. High risk operations	Low risk: 0−1 Intermediate risk: 2 High risk: 3 and above
2. Ischemic heart disease	
3. Heart Failure	
4. Cerebrovascular disease	
5. Elevated serum creatinine > 180 umol.l^–1^	
6. Hemoglobin/HbA1C ratio > 0.7	

RCRI, lee’s revised cardiac risk index.

### Demographics

**FIGURE 2:**
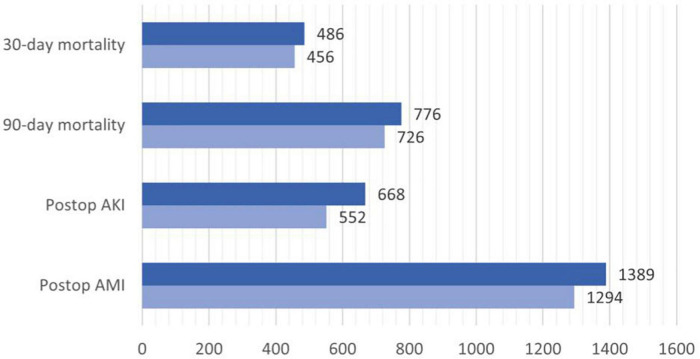
Barchart of patients in moderate + high-risk category according to RCRI (light blue) and RCRI-HH (dark blue).

**FIGURE 3:**
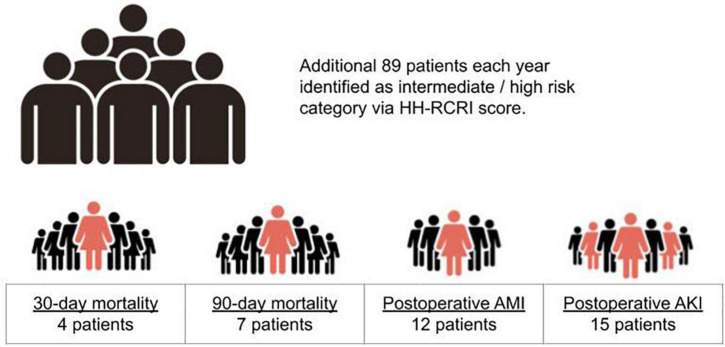
Pictorial representation of HH-RCRI score in identifying an additional 687 patients in the intermediate + high risk category.

Of the 20,099 diabetic patients, 5,421 (27.0%) patients had DM on insulin and 8,541 patients had HH ratio of >0.7. A total of 12.3% had postoperative AMI (elevated postoperative troponin of >65 ng.L^–1^) and 7.3% with postoperative AKI (KIDGO Stage 2 and above). The 30-day mortality for this cohort was 4.2 and 6.5% passed away in 90 days (Table 2). Substitution of HH ratio with the “DM on insulin” component of RCRI resulted in more patients being put in the intermediate-risk category (2,945 in RCRI group vs. 3,536 in substituted HH-RCRI score group) and high-risk category (2,114 in RCRI group vs. 2,210 in substituted HH-RCRI score group). Of those, 23 patients who were in the original intermediate and high-risk groups were moved to the low-risk group after replacement with HH ratio. In total, an additional 687 patients (average 89 patients per year) would have been put to an intermediate or high-risk category with the HH-RCRI substitution (Figure 2). Of the 89 (3.1%) additional patients identified each year, 4 (0.1%) patients had 30-day mortality, 7 (0.24%) had 90-day mortality, 12 (0.41%) patients had postoperative AMI and 15 (0.52%) patients had postoperative AKI (Figure 3).

**TABLE 2:** Patients who had 30- and 90-day mortality, postoperative acute myocardial infarction (AMI), and acute kidney injury (AKI).

Variable	Total patients	30-day mortality, *N* = 854 (4.2%)	90-day mortality, *N* = 1,313 (6.5%)	Postop AMI, *N* = 2,463 (12.3%)	Postop AKI, *N* = 1,467 (7.3%)
**RCRI components**					
Ischemic heart disease	5,159	450 (8.7%)	661 (12.8%)	1,139 (22.0%)	487 (9.4%)
Cerebrovascular disease	1,469	112 (7.6%)	196 (13.3%)	340 (23.1%)	114 (7.8%)
Congestive heart failure	1,650	213 (12.9%)	301 (18.2%)	432 (26.2%)	182 (11.0%)
DM on insulin	5,421	316 (5.8%)	500 (9.2%)	955 (18.2%)	473 (8.7%)
Creatinine > 180 umol.l^–1^	3,927	363 (9.2%)	586 (14.9%)	1,141 (29.4%)	489 (12.5%)
High risk operation	1,152	54 (4.7%)	83 (7.2%)	109 (9.5%)	128 (11.6%)
HH Ratio > 0.7	8,541	413 (4.8%)	665 (7.8%)	1,415 (17.1%)	811 (9.5%)
RCRI risk categories					
Low risk	15,040	398 (2.7%)	587 (3.9%)	1,169 (7.8%)	915 (6.1%)
Intermediate risk	2,945	190 (6.5%)	339 (11.5%)	615 (20.8%)	302 (10.3%)
High risk	2,114	266 (12.6%)	387 (18.3%)	679 (32.1%)	250 (11.8%)
**Substituted HH – RCRI score**					
Low risk	14,353	368 (2.6%)	537 (3.7%)	1,074 (7.5%)	799 (5.6%)
Intermediate risk	3,536	212 (6.0%)	356 (10.1%)	678 (19.2%)	396 (11.2%)
High risk	2,210	274 (12.4%)	420 (19.0%)	711 (32.2%)	272 (12.3%)
**n (% risk)**					

Stratified by RCRI score and substituted HH-RCRI score. RCRI, lee’s revised cardiac risk index; HH ratio, hemoglobin/HbA1C ratio; Postop, postoperative; AMI, acute myocardial injury; AKI, acute kidney injury; DM, diabetes mellitus.

The overall population was predominantly male (56%), Chinese (63%), and had BMI > 25.0 (58%). The majority of the patients underwent vascular operations (17%) and bowel operations (8.9%), and 17% of the patients had end-stage renal failure (ESRF) on dialysis. The overall median preoperative HbA1C was 7.40 (2.08) and hemoglobin of 11.8 (2.29) (Table 3).

**TABLE 3:** Patient demographics stratified by postoperative acute myocardial infarction (AMI).

Variable	Overall, *N* = 20,099	No AMI, *N* = 17,636	AMI, *N* = 2,463
Gender (female)	8,762 (44%)	7,728 (44%)	1,034 (42%)
Age	63.00 (13.34)	63.00 (13.38)	65.00 (12.89)
**Race**			
Chinese	12,702 (63%)	11,144 (63%)	1,558 (63%)
Indian	3,334 (17%)	3,012 (17%)	322 (13%)
Malay	2,948 (15%)	2,497 (14%)	451 (18%)
Others	1,115 (5.5%)	983 (5.6%)	132 (5.4%)
**BMI class**			
Normal	6,816 (39%)	5,855 (38%)	961 (43%)
Overweight (BMI > 25.)	10,312 (58%)	9,119 (59%)	1,193 (53%)
Underweight (BMI < 18.5)	573 (3.2%)	475 (3.1%)	98 (4.4%)
**ASA status**			
1 and 2	7,747 (40%)	7,338 (43%)	409 (17%)
3	11,064 (57%)	9,267 (54%)	1,797 (73%)
4	708 (3.6%)	460 (2.7%)	248 (10%)
ESRF	3,187 (17%)	2,077 (12%)	1,110 (45%)
**Type of surgery**			
Bowel operation	1,794 (8.9%)	1,569 (8.9%)	225 (9.1%)
Thoracic operation	234 (1.2%)	221 (1.3%)	13 (0.5%)
Neurosurgery	166 (0.8%)	146 (0.8%)	20 (0.8%)
Vascular operation	3,464 (17%)	2,683 (15%)	781 (32%)
**Raw RCRI score**			
0	9,122 (45%)	8,527 (48%)	595 (24%)
1	5,918 (29%)	5,344 (30%)	574 (23%)
2	2,945 (15%)	2,330 (13%)	615 (25%)
3	1,553 (7.7%)	1,119 (6.3%)	434 (18%)
4	494 (2.5%)	279 (1.6%)	215 (8.7%)
5	67 (0.3%)	37 (0.2%)	30 (1.2%)
**Raw substituted HH – RCRI score**			
0	6,800 (34%)	6,540 (37%)	260 (11%)
1	7,553 (38%)	6,739 (38%)	814 (33%)
2	3,536 (18%)	2,858 (16%)	678 (28%)
3	1,636 (8.1%)	1,157 (6.6%)	479 (19%)
4	506 (2.5%)	315 (1.8%)	191 (7.8%)
5	67 (0.3%)	26 (0.1%)	41 (1.7%)
6	1 (<0.1%)	1 (<0.1%)	0 (0%)
Preoperative HbA1C	7.40 (2.08)	7.50 (2.09)	7.30 (1.99)
Preoperative hemoglobin (g.dL^–1^)	11.8 (2.3)	12.1 (2.2)	9.7 (2.1)
HH Ratio	0.66 (0.23)	0.65 (0.22)	0.74 (0.26)
Emergency operation	6,710 (41%)	5,602 (39%)	1,108 (59%)
Duration of surgery (min)	95.00 (119.91)	95.00 (120.36)	100.00 (116.77)
Total red blood cells transfused (ml)	276.00 (25.63)	277.00 (27.79)	273.00 (20.43)
**n (%); Median (SD)**			

RCRI, lee’s revised cardiac risk index; HH ratio, hemoglobin/HbA1C ratio; AMI, acute myocardial injury; AKI, acute kidney injury.

### Outcomes analysis

The univariate analysis showed that both RCRI score and substituted HH-RCRI score were significant for all outcomes (*p* < 0.001). Substituting DM on insulin with HH ratio results in a higher odds ratio for postoperative AMI and AKI in both intermediate and high-risk categories. The predictive value for 30-day mortality was similar for both groups, while the high-risk category of substituted HH-RCRI score fared better in 90-day mortality (OR 5.79 vs. 6.82) (Table 4).

**TABLE 4:** Univariate analysis on RCRI score and substituted HH-RCRI score on mortality and postoperative outcomes.

		RCRI score [OR, (95% Cl)]	Substituted HH – RCRI score [OR, (95% Cl)]
30-day mortality	Risk categories	<0.001	<0.001
	Intermediate risk	2.54, (2.12, 3.03)	2.42, (2.04, 2.88)
	High risk	5.30, (4.50, 6.23)	5.38, (4.56, 6.33)
90-day mortality	Risk categories	<0.001	<0.001
	Intermediate risk	3.20, (2.78, 3.68)	2.88, (2.50, 3.31)
	High risk	5.52, (4.81, 6.33)	6.04, (5.26, 6.92)
Postop AMI	Risk categories	<0.001	<0.001
	Intermediate risk	2.63, (2.34, 2.95)	2.86, (2.53, 3.22)
	High risk	5.41, (4.81, 6.08)	5.58, (4.96, 6.27)
Postop AKI	Risk categories	<0.001	<0.001
	Intermediate risk	3.70, (3.40, 4.02)	3.72, (3.44,4.03)
	High risk	5.79, (5.26, 6.37)	6.82, (6.20,7.50)

Bonferroni correction: p-value is significant if *p* < 0.00625. RCRI, lee’s revised cardiac risk index; HH ratio, hemoglobin/HbA1C ratio; Postop, postoperative; AMI, acute myocardial injury; AKI, acute kidney injury.

The receiver operator curve (ROC) analysis showed that the RCRI score is moderately predictive of 30-day mortality (AUC = 0.66), 90-day mortality (AUC = 0.67), postoperative AMI (0.69), and postoperative AKI (AUC = 0.57).

Substituted HH-RCRI score results in improved sensitivity and Area under curve (AUC) for all outcomes (*p* < 0.001 in Delong’s test) (Table 5). The best predictive value of substituted HH-RCRI score is for postoperative AMI (AUC = 0.71 with a sensitivity of 0.56 and specificity of 0.75).

**TABLE 5:** Receiver operator curve (ROC) analysis of primary and secondary outcome based on RCRI score and substituted HH-RCRI score.

	AUC	*p*-value	Sensitivity	Specificity
30-day mortality		<0.001		
RCRI score	0.659		0.534	0.761
Substituted HH – RCRI Score	0.691		0.569	0.727
90-day mortality		<0.001		
RCRI score	0.669		0.553	0.769
Substituted HH – RCRI Score	0.704		0.591	0.735
Postoperative AMI		<0.001		
RCRI score	0.685		0.525	0.787
Substituted HH – RCRI Score	0.713		0.564	0.753
Postoperative AKI		<0.001		
RCRI score	0.569		0.376	0.758
Substituted HH – RCRI Score	0.622		0.455	0.728

The sensitivity and specificity are based on a cut-off of 2 (intermediate and high risk). AUC, area under curve; RCRI, lee’s revised cardiac risk index; HH ratio, hemoglobin/HbA1C ratio; Postop, postoperative; AMI, acute myocardial injury; AKI, acute kidney injury. *p*-value, Delong’s test.

**FIGURE 4:**
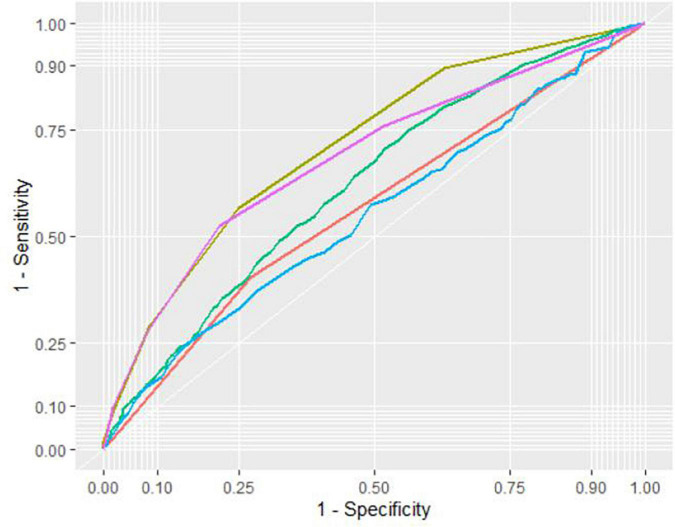
Receiver operator curve (ROC) of substituted HH-RCRI score (Yellow), RCRI score only (Purple), HbA1C:Hemoglobin ratio (Green), “DM on insulin” (Red) and preoperative HbA1C (Blue) in the prediction of postoperative acute myocardial injury (AMI).

**FIGURE 5:**
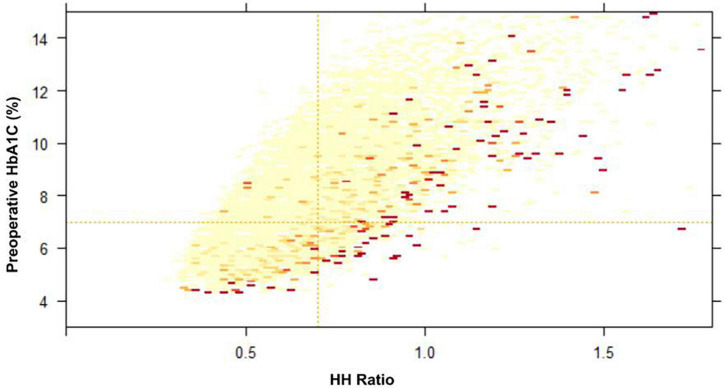
Heatmap of preoperative HbA1C and HbA1C:Hemoglobin (HH) ratio on likelihood of 90-day mortality (Higher likelihood is represented by darker color). Preoperative HbA1C cut-off of 7.0% (horizontal line) and HH ratio cut-off of 0.7 (vertical lines) is indicated.

The different predictive capacity of substituted HH-RCRI score, RCRI score, HH ratio, DM on insulin, and preoperative raw HbA1C value for postoperative AMI is represented in Figure 4. The heatmap showing the likelihood of 90-day mortality against HH ratio and Preoperative HbA1C is illustrated in Figure 5.

## Discussion

This retrospective study looked at 20,099 diabetic patients undergoing non-cardiac surgery, of which the majority were vascular and bowel operations. The mortality rates were high (4.2% 30-day mortality and 6.5% 90-day mortality) in this population. Postoperative AMI was present in 12.3% of the population and 7.3% of the patients had postoperative AKI KDIGO stage 2 and above. Substitution of the “Diabetes mellitus on insulin” component with HbA1C: hemoglobin ratio in RCRI score would put an additional 687 patients in the intermediate or high-risk category. The substituted HH-RCRI score improves the accuracy of the risk prediction model, although this is not the current standard practice. The effect is the greatest for postoperative AMI prediction.

### Using markers indicating severity of disease

The original RCRI score component includes four components from the patient’s past medical history, none of which reflect the severity of the disease. The patient could vary widely on the spectrum. This may have accounted for the lack of consistent validation of RCRI scoring in subsequent studies, where RCRI was only found to be moderately discriminating with a median AUC of 0.62 (2).

The rationale for replacing the “Diabetes Mellitus on Insulin” with HbA1C:Hemoglobin is to provide a more objective severity marker. In the original RCRI score, data only showed trending significance for “DM on insulin” for major cardiac complications in univariate analysis (3/59 patients, 5%; relative risk 2.4; 95% CI 1.2, 4.8) (1). Bearing in mind that the score was created more than a decade ago, this component of the score could have potentially reduced the sensitivity and accuracy of the scoring system.

HbA1C has become the standard screening test for diabetes in elective surgery patients in the last decade (22). In our tertiary institution, HbA1C is routinely done for all diabetic patients going for elective operations if they are not done in the last 3 months, or at the surgeon’s discretion when the patient is going for a major operation. In recent years, HbA1C is also becoming a standard preoperative test for cardiac patients (16). In emergency operations, patients often have HbA1C done when they are admitted for an infective-related process such as abscesses, and skin and soft tissue infections. This is to pick up undiagnosed DM in this group of patients and start appropriate treatment. As such, we expect to see more patients, especially diabetic ones, to have preoperative HbA1C available for risk scoring.

With the increasing use of HbA1C in the preoperative setting, the lack of its incorporation into risk scoring systems might have undermined its utility. One reason for the hesitance to incorporate HbA1c into predictive scoring could be due to an inconsistent correlation with postoperative outcomes (7–10). It has been proposed that patients who are anemic may have a falsely elevated HbA1C value due to the increased duration of circulating erythrocytes in iron deficiency. In the anemic population, HbA1C may therefore be inaccurate. The HbA1C:Hemoglobin ratio has been previously proposed to be associated with mortality and morbidity in cardiac operations (16). The rationale is to correct HbA1C for hemoglobin. It also highlights the double threat in patients who have both anemia and elevated HbA1C. Taking advantage of the increasingly available preoperative HbA1C and hemoglobin, we are able to obtain the HH ratio for most diabetic patients going for operations.

### Subjective vs. objective component in scoring systems

Historically, “DM on insulin” has been used in RCRI as an arbitrary marker of severity of diabetes. However, the actual intention may not be as such. The decision to put the patient on insulin may vary widely on the patient’s acceptance to subcutaneous injections and threshold for the primary provider to prescribe insulin. It may also vary from institutional practices, and does not account for patient’s compliance to medications. The substitution of HH ratio with “DM on insulin” would hence be more reflective of the severity of the disease.

The additional benefit of objective measurement from blood results is that the results can be pulled automatically from the system. Patients who are on medications given by private doctors will not have the insulin medications reflected on the system and will require the anesthetist to do a preoperative assessment to manually select the checklist. In our database, a total of 5,421 patients had been checked for “DM on insulin” in the RCRI risk scores. An additional 159 patients had insulin prescribed in the perioperative medications, even though the “DM on insulin” was selected to be negative. The disagreement shows the real-world problem of manual data entry. Automated charts pulled directly from the latest results would bypass this error and improve accuracy for the extraction of medical information for large data research (23).

### Prediction of postoperative outcomes

The modified RCRI score commonly used looks at pooled outcomes of 30-day mortality, myocardial infarction, and cardiac arrest (24). Duceppe et al. looked at 5 high-quality external validations and the updated risks-percentage in each group was higher than that originally described in the 1990 study. The composite of 30-day mortality, risk of postoperative AMI, and cardiac arrest was found to be 3.9, 6.0, and 10.1%, respectively, in low, intermediate, and high-risk groups. Our cohort of diabetic-only patients including both elective and emergency surgeries provides a population with higher risks. Patients who were in RCRI or substituted HH-RCRI intermediate-risk group had a 6.0−6.5% incidence of 30-day mortality and the high-risk group had 12.4−12.6%. Up to 32% of the patients in the high risks group had postoperative AMI.

The substitution of HH ratio in the HH-RCRI score was able to pick up an additional 687 patients into the intermediate to high-risk category. Of those patients, the modified score is able to identify a range of 30 to 116 patients who would have had mortality, postoperative AMI, or AKI. This number will rise as more patients have preoperative HbA1C done in the preoperative setting and more patients become eligible for the modified score.

### Postoperative acute myocardial infarction

The substituted HH-RCRI score shows the best predictive capacity for postoperative AMI (OR 5.58 (4.96, 6.27), *p* < 0.001, AUC 0.713). The postoperative AMI was detected using positive symptoms or clinical indications and high-sensitivity troponin-T in our study, which would be more sensitive than the creatine kinase muscle and brain isoenzyme (CK-MB) used in the original RCRI study. Diabetic patients who have a substituted HH-RCRI score in the intermediate-risk group have a 19.2% risk of developing postoperative AMI, and 32.2% if they are in the high-risk group. Although the outcome in our study did not study myocardial injury after non-cardiac surgery (MINS) (25), it would be a surrogate marker for patients who are at high risk of MINS. Hence, in addition to being a valuable preoperative risk prediction tool, the HH-RCRI score could be used as a guide for patient populations where routine postoperative high-sensitivity troponins should be done. This is especially important in the diabetic populations where postoperative AMIs are common, but yet could be asymptomatic and masked by analgesia.

### Use of HbA1C: Hemoglobin ratio (HH ratio)

The predictive value of preoperative HbA1C is poor compared to HbA1C: Hemoglobin ratio (Figure 5). This is consistent with a previous study in the cardiac population (16), where the HH ratio was found to have a stronger association with postoperative outcomes. To improve the accuracy of prediction, the HH ratio was adopted instead of preoperative HbA1C alone. The ratio not only corrects HbA1C for anemia but also is an expression of demand and supply imbalance. Hyperglycemia may enhance the production of free radicals and induce oxidative stress and accounts for many of the micro-and macrovascular complications from diabetes (26). Hemoglobin reflects oxygen-carrying capacity and delivery. The HH ratio was previously validated in cardiac populations where anemia was common. In this diabetic cohort going for non-cardiac surgery, the median hemoglobin was 11.8 g.dL^–1^. This highlights the usefulness of using HH ratio instead of HbA1C or hemoglobin alone in populations where anemia (defined by preoperative Hemoglobin <13.0 g.dL^–1^) is common.

### Future work

The current RCRI score was developed in 1999, and medical practices have changed drastically since then. Although there are many better scoring systems such as NSQIP, the RCRI score still serves a function of being a quick and easy screening tool. Replacing components of the score with objective severity markers is worth looking into. For instance, using Pro-BNP or presence of diastolic or systolic heart failure on 2D echo instead of “History of heart failure.” These investigation-guided severity markers more accurately reflect the severity of disease on a continuous variable, rather than “Yes and No.” In addition, this reduces the subjectivity of operator dependence and would allow for more consistent risk scoring across cohorts. We may hope to achieve a more accurate modified-RCRI score in the future as these laboratory markers are done more frequently in the modern era of medicine.

The study also provided an additional affirmation of the predictive value of the HH ratio even in the non-cardiac population. Further studies to validate the ratio in other cohorts such as major abdominal operations or hip surgeries could be considered. The value of the HH ratio as a preoperative optimization target can also be explored in clinical trials.

## Limitations

Our study was a retrospective one and contains some of the problems inherent in such studies. This is a single-centered study, and there might be patient selection bias. The decision to perform HbA1C was left to the surgeon and anesthetist’s decision, and the cohort included in the final analysis may be biased. A total of 17,031 patients were excluded from the study as no HbA1C was done. HbA1C was recommended as a routine preoperative test for all diabetic patients if they do not have one done within the last 3 months. This recommendation was only implemented in the institution after 2018. The study excluded all non-diabetic patients for this reason.

The outcome measurement of Postoperative AMI is defined by elevated high-sensitivity Troponin-T from postoperative laboratory values, with the assumption that patients who had troponin done would have had other clinical findings that are suspicious of myocardial ischemia. This would fulfill the diagnostic criteria based on ESC guidelines (27). As postoperative troponin-T is not done for all patients, the incidence of postoperative AMI may be under-estimated. Postoperative MINS could not be studied as an outcome as troponin-T was not done in everyone in this retrospective cohort. Data for other cardiac events such as arrhythmias are not accounted for.

## Conclusion

Although not currently a standard practice, substitution of the “DM on insulin” component with HbA1C:Hemoglobin ratio (HH Ratio) in RCRI score improves the accuracy of the RCRI risk prediction model in diabetic patients going for non-cardiac surgery.

## Data availability statement

The data analyzed in this study is subject to the following licenses/restrictions: Single centered dataset. Requests to access these datasets should be directed to YK, yuhe.ke@mohh.com.sg.

## Ethics statement

The studies involving human participants were reviewed and approved by SingHealth Centralised Institutional Review Board (CIRB Reference number: 2020/2915). Ethics review and approval/written informed consent was not required as per local legislation and institutional requirements.

## Author contributions

YK: conceptualization of the manuscript, statistics, and drafting the manuscript. NS: statistics and consultation. HA: advisory role to the manuscript. All authors contributed to the article and approved the submitted version.
